# An Audit to Assess Compliance With DVLA Guidelines on a Mental Health Rehabilitation Unit

**DOI:** 10.1192/bjo.2024.618

**Published:** 2024-08-01

**Authors:** Olusegun Popoola, Ranjan Baruah, Sion Gilbey, Oliver Pentz

**Affiliations:** Mersey Care NHS Foundation Trust, Liverpool, United Kingdom

## Abstract

**Aims:**

Patients with serious mental disorders like psychosis may pose a significant risk to themselves and others when they drive. The DVLA has set out guidance for driving for patients with psychiatric disorders, substance use disorders, and for those taking psychotropic medications. It's good medical practice to identify risks associated with driving, discuss, advise appropriately, and document the same in the clinical notes.

To assess the compliance of the mental health professionals at Rathbone Rehabilitation Centre (RRC) with DVLA guidelines regarding patients about driving restrictions, documenting this appropriately and to increase awareness of the DVLA guidelines.

**Methods:**

Data of all the discharged patients from RRC over a 12-month period was collected following a standardised process and assessed for 6 parameters.

A total of 51 discharges were identified and audited against the DVLA guidelines.

**Results:**

51 (100%) patients had a mental health diagnosis documented on patient electronic records (Rio).

9 (18%) of patients had their driving status documented. 42 (82%) did not.

Of the 9 patients whose driving status was recorded, 6 did not drive and are thus labelled not applicable for subsequent criteria. The type of vehicle driven was not documented in any of the cases and therefore was 0%.

Of the 3 patients who drive, 2 (67%) had been informed that their condition may affect their ability to drive.

67% had documented evidence of receiving advice on driving restrictions.

67% had documented evidence that the practitioner has informed the patient that they have a legal duty to inform the DVLA about their condition.

**Conclusion:**

An action plan was designed to improve compliance with DVLA guidelines for practitioners managing inpatients.

- On admission all patients should be asked for their driving status and the result documented on Rio. This could be done on the clerking admissions proforma on Rio.
- For all patients that do drive, the types of vehicles they drive should be documented–this can also be included in the clerking admissions proforma on Rio.
- At their first ward review/discharge meeting and whenever relevant, patients should be informed whether their condition affects their ability to drive and if so, what the restrictions are. They should be informed of the legal requirements regarding informing the DVLA and documented.
- To consider driving status when assessing risk.

